# Goal-Directed Fluid Therapy Using Stroke Volume Variation Does Not Result in Pulmonary Fluid Overload in Thoracic Surgery Requiring One-Lung Ventilation

**DOI:** 10.1155/2012/687018

**Published:** 2012-06-21

**Authors:** Sebastian Haas, Volker Eichhorn, Ted Hasbach, Constantin Trepte, Asad Kutup, Alwin E. Goetz, Daniel A. Reuter

**Affiliations:** ^1^Department of Anesthesiology, Center of Anesthesiology and Intensive Care Medicine, Cardiovascular Research Center, University Medical Center Hamburg-Eppendorf, Martinistra**β**e 52, 20246 Hamburg, Germany; ^2^Department of Anesthesiology and Intensive Care, Evangelisches Krankenhaus Mühlheim, 45468 Mühlheim, Germany; ^3^Department of General, Visceral and Thoracic Surgery, University Medical Center Hamburg-Eppendorf, 20246 Hamburg, Germany

## Abstract

*Background*. Goal-directed fluid therapy (GDT) guided by functional parameters of preload, such as stroke volume variation (SVV), seems to optimize hemodynamics and possibly improves clinical outcome. However, this strategy is believed to be rather fluid aggressive, and, furthermore, during surgery requiring thoracotomy, the ability of SVV to predict volume responsiveness has raised some controversy. So far it is not known whether GDT is associated with pulmonary fluid overload and a deleterious reduction in pulmonary function in thoracic surgery requiring one-lung-ventilation (OLV). Therefore, we assessed the perioperative course of extravascular lung water index (EVLWI) and p_a_O_2_/F_i_O_2_-ratio during and after thoracic surgery requiring lateral thoracotomy and OLV to evaluate the hypothesis that fluid therapy guided by SVV results in pulmonary fluid overload. *Methods*. A total of 27 patients (group T) were enrolled in this prospective study with 11 patients undergoing lung surgery (group L) and 16 patients undergoing esophagectomy (group E). Goal-directed fluid management was guided by SVV (SVV < 10%). Measurements were performed directly after induction of anesthesia (baseline—BL), 15 minutes after implementation OLV (OLVimpl15), and 15 minutes after termination of OLV (OLVterm15). In addition, postoperative measurements were performed at 6 (6postop), 12 (12postop), and 24 (24postop) hours after surgery. EVLWI was measured at all predefined steps. The p_a_O_2_/F_i_O_2_-ratio was determined at each point during mechanical ventilation (group L: BL-OLVterm15; group E: BL-24postop). *Results*. In all patients (group T), there was no significant change (*P* > 0.05) in EVLWI during the observation period (BL: 7.8 ± 2.5, 24postop: 8.1 ± 2.4 mL/kg). A subgroup analysis for group L and group E also did not reveal significant changes of EVLWI. The p_a_O_2_/F_i_O_2_-ratio decreased significantly during the observation period (group L: BL: 462 ± 140, OLVterm15: 338 ± 112 mmHg; group E: BL: 389 ± 101, 24postop: 303 ± 74 mmHg) but remained >300 mmHg except during OLV. *Conclusions*. SVV-guided fluid management in thoracic surgery requiring lateral thoracotomy and one-lung ventilation does not result in pulmonary fluid overload. Although oxygenation was reduced, pulmonary function remained within a clinically acceptable range.

## 1. Introduction

Early, preemptive strategies of hemodynamic optimization are an important factor for sufficient organ microcirculation and are considered to be associated with reduced morbidity and mortality [1]. Within this context, improvement of intravascular volume status seems essential. After central venous pressure (CVP) was identified as inappropriate for the assessment of intravascular volume status, volumetric and functional parameters of preload came into the focus of interest. Clinical studies were thus initiated to evaluate the potential of fluid management guided by these parameters. Although one study failed to demonstrate improvements, other studies have shown improvements in hemodynamics leading to benefits in clinical outcome when goal-directed fluid therapy was guided by global enddiastolic volume (GEDV), pulse pressure variation (PPV), or stroke volume variation (SVV) [2–5]. However, in thoracic surgery, the potential benefit or even disadvantage of volume management guided by functional parameters of preload have not been evaluated thus far. One possible disadvantage may be the fact that fluid management guided by functional parameters of preload is suspected to be a rather fluid aggressive approach since in the clinical studies the goal-directed group received more fluids than the control group [2–5]. Lopes and colleagues reported a more than double amount of fluid administration in the PPV-guided fluid management group [4]. It hast to be kept in mind that pulmonary fluid overload has been identified as an independent risk factor for the development of perioperative acute lung injury after lung surgery [6]. Therefore, a volume restrictive regime is usually recommended for lung surgery [6–9]. 

The extravascular fluid content of the lungs can be quantified using transpulmonary thermodilution (TCPTD) by measuring the extravascular lung water index (EVLWI). EVLWI by thermodilution was identified as more sensitive for quantifying the fluid content of the lungs than chest radiographs [10–12]. Furthermore, EVLWI is known as a prognostic parameter for clinical outcome in critically ill patients [13–17]. 

Fluid management in thoracic surgery is of particular importance because of the influence of one-lung ventilation (OLV). It has been reported that OLV by itself can be a cause of postoperative pulmonary edema [17–20]. Edema formation after OLV is explained by oxidative stress during—and in particular immediately following—OLV by reexpansion of the deflated lung, once conventional ventilation is reestablished [19–22]. Therefore, OLV might act as an additional factor for aggravating a perioperative pulmonary fluid overload.

In open chest conditions, the use of functional parameters of preload such as SVV to predict volume responsiveness is controversial [23–28]. As this question is not conclusively answered, the feasibility of SVV-guided fluid management remains unclear. 

The hypothesis of our study was that fluid management guided by SVV results in fluid overload of the lungs during and after thoracic surgery that requires lateral thoracotomy and OLV. Therefore, the aim of the study was to investigate the influence of SVV-guided fluid management on the perioperative course of the formation of pulmonary extravascular fluid content as measured by EVLWI (first endpoint) and gas exchange measured by p_a_O_2_/F_i_O_2_-ratio (second endpoint) in thoracic surgery requiring lateral thoracotomy and OLV. Furthermore, 30-day mortality was assessed.

## 2. Materials and Methods

Approval for this study was provided by the Ethics Committee of the Hamburg Medical Board (Aerztekammer Hamburg). All patients gave written informed consent.

### 2.1. Patients

A total of 27 patients (group T, *n* = 27) scheduled for elective thoracic surgery requiring OLV were enrolled in this prospective study. Exclusion criteria were age under 18 years, cardiac arrhythmias and/or atrial fibrillation, and the presence of contraindications to femoral arterial catheterization.

### 2.2. Anesthesia

All patients were premedicated with midazolam 0.1 mg/kg orally before arriving in the operating room. All patients received an epidural catheter at level Th4 to Th7. A bolus of 0.125 mL/kg of 0.5% bupivacaine and 10 *μ*g sufentanil was administered followed by a continuous administration of 0.5% bupivacaine (0.05 mL/kg/h). After surgery, administration of bupivacaine was stopped and 0.2% ropivacaine was administered at an infusion rate of 8 mL/h and was adjusted to the clinical situation. Directly after placement of the epidural catheter, general anesthesia was induced with 0.7 *μ*g/kg sufentanil, 2.5 mg/kg propofol, and 0.6–0.8 mg/kg rocuronium. Anesthesia was maintained with isoflurane 1–1.5% in oxygen and sufentanil, and rocuronium was used for further relaxation. During surgery, a dosage of 0.2-0.3 *μ*g/kg sufentanil was administered every 45 minutes or when clinically required. Neuromuscular monitoring was used for further rocuronium application, and a dosage of 0.1-0.2 mg/kg rocuronium was repeated when the train of four (TOF) ratio was >0.5. A left-sided double-lumen tube (Broncho-Cath; 37–41 French, Mallinckrodt Medical Ltd, Ireland) was introduced and adjusted using a fiberoptic bronchoscope.

### 2.3. Ventilation

Ventilation was performed using pressure-controlled mode. During conventional ventilation of both lungs, pressure control was adjusted to achieve tidal volumes of 8 mL/kg. A positive end-expiratory pressure (PEEP) of 8 cm H_2_O was selected. The inspiratory-expiratory ratio was 1 : 1.7. The respiratory rate was adjusted to maintain the arterial partial pressure of carbon dioxide between 36 and 44 mmHg. During OLV, the ventilation pattern was modified using a PEEP of 3 cm H_2_O, and tidal volumes of 4–6 mL/kg. The inspired oxygen fraction was initially 1.0 and decreased to a level which allowed maintenance of  P_a_O_2_ > 80 mmHg.

### 2.4. Hemodynamic Monitoring

A central venous line was placed into the internal jugular vein for the continuous monitoring of central venous pressure, drug administration and injection of cold indicator for thermodilution. A 5-Fr thermistor-tipped catheter (PICCO, PV2025L20, Pulsion Medical Systems AG, Munich, Germany) was inserted into the femoral artery and connected to a hemodynamic monitor (PiCCO plus, Pulsion Medical Systems AG, Munich, Germany) for continuous measurement of SVV, arterial pressure and intermittent assessment of cardiac index (CI), global enddiastolic volume index (GEDI), stroke volume index (SVI), and EVLWI by TCPTD. Thermodilution measurements were performed by three sequential central venous injections of 10 mL cold saline solution (<8°C). All thermodilution curves were examined, and measurements were accepted if none of the three consecutive values differed by more than 10% from the mean.

### 2.5. Study Protocol

All patients received continuous infusion of crystalloid infusion (Sterofundin Ecoflac Plus, Braun AG, Melsung Germany) at a rate of 9 mL/kg/h intraoperatively. Continuous administration was reduced to 4 mL/kg/h after surgery and continuous administration was further reduced to 2 mL/kg/h after extubation. These rather high infusion rates were chosen to provide a rather fluid aggressive study protocol. 1000 mL Sterofundin content 5.5 g NaCl, 0.3 g KCl, 0.37 g CaCl_2_, 0.2 g MgCl_2_, and 0.05 g natrium-lactate. Osmolarity is 299 mOsm/L. Additionally, a bolus of 5 mL/kg colloid (Voluven 130/0.4 6%, Fresenius Kabi AG, Bad Homburg, Germany) was given when SVV was above 10% and repeated if necessary until SVV returned to below 10%. Colloid administration, in order to achieve an SVV of lower than 10% was primarily done prior to open-chest conditions and continued during the surgical phase with a laterally opened thoracic cavity and after surgery. If clinically indicated (according to the International Normalized Ratio (INR) > 1.6 in combination with active bleeding), the required fluid loading was done with fresh frozen plasma. When mean arterial pressure dropped below 60 mmHg despite fluid resuscitation or during sudden blood loss, continuous norepinephrine administration was initiated. After extubation, SVV-guided fluid management was discontinued because SVV is not validated for use during spontaneous breathing. Hemodynamic measurements as well as arterial blood gas analyses were performed after induction of anesthesia (baseline—BL), 20 min after implementation of OLV (OLVimpl15) and 15 min after termination of OLV (OLVterm15), as well as at 6 h (6postop), 12 h (12postop), and 24 h (24postop) after the end of surgery.

### 2.6. Surgical Procedures

In the 11 patients with lung surgery, lobectomy was performed in 9 patients and bi-lobectomy in 2 patients. In the esophageal surgery group all 16 patients underwent transthoracic esophagectomy with two-field lymphadenectomy and reconstruction achieved by gastric pull up. None of the patients received neoadjuvant treatment.

### 2.7. Extravascular Lung Water Index (EVLWI) and Pulmonary Function

EVLWI was measured by transpulmonary thermodilution for quantification of pulmonary extravascular fluid content. To evaluate pulmonary compliance, the static pulmonary compliance  *C*  [L/cmH_2_O] was assessed using the equation *C* = *V*/Δ*P*, where *V* is the tidal volume and Δ*P* is the difference of inspiration pressure and PEEP. Oxygenation was quantified by calculating the p_a_O_2_/F_i_O_2_-ratio (arterial partial pressure of oxygen p_a_O_2_/inspiratory oxygen concentration F_i_O_2_) for each patient at each point of measurement as long as the patient was intubated and mechanically ventilated (Group L: from BL to OLVterm15, Group E: from BL to 24postop).

### 2.8. Metabolic and Hemodynamic Parameters

Blood lactate, central venous oxygen saturation (ScvO_2_), and base excess (BE) were assessed. Furthermore, hemodynamic data such as mean arterial pressure (AP_mean_), central venous pressure (CVP), global enddiastolic volume index (GEDI), stroke volume index (SVI), cardiac index (CI), pulmonary compliance, and norepinephrine administration were recorded at the predefined steps BL-24postop to describe metabolic and hemodynamic consequences during and after SVV-guided fluid management in thoracic surgery requiring lateral thoracotomy and OLV.

### 2.9. Subgroup Analysis

To further evaluate our hypothesis, a subgroup analysis of two subgroups was performed. Firstly, the course of EVLWI was explored in the lung surgery (group L: *n* = 11). Lung surgery is usually associated with a relatively short period of OLV and direct trauma to the lungs. Secondly, the course of EVLWI was investigated in transthoracic esophagectomy (group E: *n* = 16), an intervention involving severe general surgical trauma, a longer period of OLV, and higher fluid turnover.

### 2.10. Statistical Analysis

Descriptive statistical analysis was performed using SigmaStat and SigmaPlot (Systat Software, Inc., Germany). Student  *t*-test was performed for corresponding group comparison regarding patients characteristics and surgery data between group L and group E. Normally distributed data (Kolmogorov-Smirnov-Test) were analyzed with a Tukey's one-way analysis of variance for repeated measurements (ANOVA), nonnormally distributed parameters were analyzed with Kruskal-Wallis Analysis of Variance (ANOVA) on Ranks. Results are given as mean ± standard deviation (SD). A  *P*  value < 0.05 was considered statistically significant.

## 3. Results

Patient's characteristics and comorbidities are given in Table 1. Patients did not differ significantly regarding age, body mass index, and ASA classification. Data regarding surgery, fluid administration and diuresis are presented in Table 2. All parameters were significantly higher in group E than in group L (*P* < 0.05) apart from the duration of OLV (*P* = 0.057) and red blood cell administration (*P* = 0.223).

### 3.1. Extravascular Lung Water Index (EVLWI)

In all patients (group T), EVLWI did not change significantly during the observation period (BL: 7.8 ± 2.5, 24postop: 8.1 ± 2.4 mL/kg). The course of EVLWI in the subgroup analysis (group L: BL: 7.9 ± 1.7 mL × kg^−1^, 24postop: 7.2 ± 1.9 mL/kg; group E: BL: 7.8 ± 3 mL/kg, 24postop: 9.1 ± 2.5 mL/kg) also revealed no significant changes. The highest mean of EVLWI was measured in group E at 24postop (9.1 mL/kg).

### 3.2. P_a_O_2_/F_i_O_2_-Ratio

In all patients (group T), the p_a_O_2_/F_i_O_2_-ratio decreased when comparing values prior to (BL) and after OLV (OLVterm15) (BL: 419 ± 122 mmHg, OLVterm15: 334 ± 92 mmHg). In subgroup L, the p_a_O_2_/F_i_O_2_-ratio also decreased significantly from 462 ± 140 mmHg at BL to 338 ± 112 mmHg at OLVterm15. In group E, a decrease in the p_a_O_2_/F_i_O_2_-ratio was observed 24 hrs after surgery (BL 389 ± 101 mmHg, 24postop: 303 ± 74 mmHg). The lowest mean p_a_O_2_/F_i_O_2_-ratio was observed in group E except for during OLV at timepoint 24postop (303 ± 74 mmHg). Patients of group L were extubated immediately after the end of surgery, whereas patients of group E were extubated 24 h after the end of surgery.

### 3.3. Cardiac Index

CI was increased at timepoints OLVimpl15 and 6postop compared to baseline timepoint BL in all patients. (BL: 2.8 ± 0.9 L/min/m^2^, OLVimpl15: 3.9 ± 0.9 L/min/m^2^, 6postop: 3.5 ± 0.9 L/min/m^2^). In the subgroup analysis, CI increased significantly at OLVimpl15 in group E (BL: 2.7 ± 0.9 L/min/m^2^, OLVimpl15: 3.7 ± 1 L/min/m^2^).

### 3.4. 30-Day Mortality

One patient in group L died due to malignoma-induced erosive bleeding of the pulmonary artery on the second day after surgery. One patient in group E died 28 days after surgery due to septic shock and severe mediastinitis. Thus, 30-day mortality was 7.4% for all patients, 9.1% in group L, and 6.3% in group E.

### 3.5. Metabolic Data

Lactate levels, central venous oxygen saturation (ScvO_2_), base excess (BE), and hemoglobin (Hb) are given in Table 3. Lactate levels increased significantly at 6postop, 12postop, and 24postop in group T and group E compared to BL. However, levels of lactate remained very low (<1.4 mmol/L). ScvO_2_ decreased significantly, in all groups at 12postop, and 24postop compared to BL, but always remained in a range above 70%. BE decreased significantly at 6postop, 12postop and 24postop compared to BL. Hb was also significantly reduced at most timepoints compared to BL. At this point it has to be clearly stated that all significant changes in all metabolic data were well within normal values and have to been seen clinically irrelevant.

In addition to EVLWI, p_a_O_2_/F_i_O_2_-ratio and CI, further data on hemodynamics (AP_mean_ [mmHg], CVP [mmHg], GEDI [mL/m^2^]), SVI [mL/m^2^], pulmonary compliance [L/cmH_2_O], and norepinephrine administration [*μ*g/kg/min] are shown in Table 4. In group L, 6 of 11 patients required norepinephrine administration temporarily; in group E, all patients required temporary norepinephrine administration.

## 4. Discussion

Although fluid management guided by functional parameters of preload are suggested to be rather fluid aggressive and validity of these parameters are controversial under open chest conditions, the present study shows that this goal-directed approach does not result in pulmonary fluid overload and deleterious reduction of pulmonary function in thoracic surgery requiring lateral thoracotomy and OLV. Furthermore, no derangement in metabolic parameters or increase in mortality associated with an altered pulmonary function could be identified. Whether this treatment strategy that demonstrated potential clinical benefit in abdominal and cardiac surgery is also potentially useful in this field of surgery cannot clearly be stated. However, incurring an increased risk of significant pulmonary fluid overload or critical reduction in pulmonary function seems not to be a clinically relevant problem.

In thoracic surgery, esophagectomy and lung surgery are counted amongst the most commonly performed surgical operations. In esophagectomy patients, clinical trials have demonstrated an increase in EVLWI [29, 30]. Oshima and colleagues reported values of EVLWI > 10 mL/kg perioperatively after esophagectomy without having a standardized protocol for fluid administration. Severe surgical trauma, lymphatic node extirpation, and systemic inflammatory response are seen as the main causes for this increase in EVLWI. Furthermore, OLV might also contribute to the development of a postoperative pulmonary edema, since it is known that OLV is correlated with pulmonary oxidative stress [21, 22]. In our study using SVV for guidance of fluid therapy, no significant increase of EVLWI in the subgroup of esophagectomy was found perioperatively and levels of EVLWI remained below 10 mL/kg.

In the patients that underwent lung surgery, EVLWI was even lower and did not exceed values of 9 mL/kg. However, the validity of EVLWI in lung surgery must be interpreted with caution, particularly if lung tissue is resected, as was the case in our study where pulmonary lobectomy and bi-lobectomy were performed. Basically, EVLWI is underestimated when lung tissue is resected because any decrease in pulmonary blood volume induced by lung tissue resection influences the intrathoracic blood volume. Since EVLWI is calculated as the difference between intrathoracic thermal volume and intrathoracic blood volume—which in this instance would be overestimated—EVLWI is underestimated following lung tissue resection [31, 32]. Therefore, the results of EVLWI in the lung surgery group might be regarded as artificially low. Even if EVLWI was underestimated by 20%, EVLWI would not exceed the maximum of 11 mL/kg as seen in the lung surgery group (the highest value of measurement at OLVTERM15) and not 9 mL/kg 24 hours after surgery. This level is still within a clinically acceptable range when related to the results of Sakka and coworkers who described an EVLWI > 12 mL/kg to be correlated with a worse outcome in critically ill patients [13].

Volume deficiency indicated by SVV was corrected using colloid infusion in our study. Crystalloid was administered only at a maintenance rate. Since it is known that, compared to colloids, only one fifth of the intravenously infused volume of crystalloids remains within the intravascular space, it must be assumed that a strategy based on a protocol using crystalloids exclusively would potentially have led to a higher EVLWI value and potentially a more pronounced deterioration in pulmonary function [33].

Gas exchange was reduced in the lung surgery group and in the esophagectomy group. In lung surgery, a decrease in the p_a_O_2_/F_i_O_2_-ratio can be explained by resection of lung tissue leading to a reduction of the alveolar surface necessary for gas exchange. In patients undergoing esophagectomy, postoperative deterioration of gas exchange (such as a decrease in the p_a_O_2_/F_i_O_2_-ratio) is common and clinically challenging [34–37]. However, the decrease in the p_a_O_2_/F_i_O_2_-ratio was only moderate and values always remained above 300 mmHg. Therefore, SVV-guided volume therapy seemed not to have aggravated this clinical problem.

After initiation of SVV-guided fluid management, CI was increased at two timepoints compared to the baseline measurement. Although the increase did not reach statistical significance at all timepoints, these data suggest that SVV-guided fluid management contributes to an improved CI even in open chest thoracic surgery, being the basis for optimization of tissue oxygenation.

GEDI did not change significantly during the observation period. This fact provides evidence that the volume replacement strategy oriented to SVV led to a stable preload condition in these patients. A comparison group with more restrictive fluid management would have been desirable at this point and certainly, the lack of this comparison group remains the major limitation of this study. Other limitations have to be taken into consideration. Only the total amount of fluid administration 24 hours after surgery was recorded, and thus fluid administration cannot be differentiated according to the time line BL-24postop. The validity of SVV and the validity of transpulmonary thermodilution parameters during OLV have not been explored in detail. Thus far only one clinical study has shown SVV to be a predictor for volume responsiveness during OLV [38]. Furthermore, after thoracotomy with open chest conditions, SVV is not without controversy regarding prediction of volume responsiveness [23–28]. However, our data has revealed that even if SVV-guided fluid management is not definitively validated under open chest conditions and OLV, severe pulmonary fluid overload is not inevitable.

Our study was not designed to demonstrate the clinical advantage of a SVV-guided fluid management in comparison to a control group. Furthermore, it is difficult to comment on any real safety in a study with a limited number of participants included, particularly when there is no comparison group. Therefore, our results have to be interpreted with caution. Nevertheless, our study forms the basis for further investigation regarding SVV-guided fluid management in thoracic surgery requiring open chest conditions and OLV, which has previously been effectively performed in other fields of surgery.

## Figures and Tables

**Table 1:** Patient's characteristics and comorbidities. Patients did not differ significantly (*P* > 0.05) between group L and group E regarding age, body mass index, and ASA classification.

	All patients (*n* = 27)	Group L (*n* = 11)	Group E (*n* = 16)	*P* value
Age [years]	61.3 ± 11.6	62.1 ± 10.6	60.4 ± 13.4	*P* = 0.72
Body mass index [kg/m^2^]	25.4 ± 5.2	24.7 ± 5.5	26 ± 5.2	*P* = 0.56
ASA classification	2.7 ± 0.3	2.5 ± 0.3	2.8 ± 0.3	*P* = 0.65
Coronary artery disease	5	2	3	
Impaired ventricular function (EF < 40%)	3	1	2	
Renal insufficiency	4	2	2	
Chronic obstructive pulmonary disease	10	8	2	

**Table 2:** Surgical data, fluid administration, and diuresis. ^∗^Statistical significance between group L and group E analyzed by students *t*-test (*P* < 0.05).

	All patients (*n* = 27)	Group L (*n* = 11)	Group E (*n* = 16)	*P* value
Duration of surgery [min]	294.3 ± 144.4	177.5 ± 76.7	375.7 ± 123.6^∗^	*P* < 0.001
Duration of OLV [min]	134.8 ± 25.6	113.3 ± 55.7	149.3 ± 39.3	*P* = 0.057
Blood loss during surgery [mL]	538 ± 784	190 ± 347	778 ± 914	*P* = 0.037
Number of patients received noradrenaline	22	6	16	
Crystalloid administered during observation period [mL/kg/h]	3.4 ± 0.8	2.8 ± 0.8	3.8 ± 0.8^∗^	*P* = 0.003
Colloid administered during observation period [mL/kg/h]	1.2 ± 0.4	0.6 ± 0.3	1.5 ± 0.5^∗^	*P* < 0.001
Fresh frozen plasma administered during observation period [mL]	514 ± 840	0	770 ± 933^∗^	*P* = 0.012
Packed red blood cells administered during observation period [mL]	545 ± 697	320 ± 345.1	640 ± 642	*P* = 0.223
Diuresis [mL/kg/h]	1.3 ± 0.4	0.9 ± 0.3	1.6 ± 0.4^∗^	*P* < 0.001

**Table 3:** Metabolic data. ScvO_2_: central venous oxygen saturation; BE: base excess. ^∗^Difference to BL in analysis of variance (ANOVA) (*P* < 0.05). BL: directly after induction of anesthesia; OLVimpl15: 15 minutes after beginning OLV; OLVterm15: 15 minutes after cessation of OLV; 6postop: 6 hours after surgery; 12postop: 12 hours after surgery; 24postop: 24 hours after surgery.

	BL	OLVimpl15	OLVterm15	6postop	12postop	24postop
Lactate_T_ [mmol/L]	0.8 ± 0.3	0.8 ± 0.2	1.1 ± 0.5	1.2^∗^ ± 0.6	1.1^∗^ ± 0.4	1.2^∗^ ± 0.4
Lactate_L_ [mmol/L]	0.8 ± 0.4	0.7 ± 0.2	1 ± 0.5	0.9 ± 0.3	1 ± 0.4	1.1 ± 0.4
Lactate_E_ [mmol/L]	0.8 ± 0.2	0.9 ± 0.2	1.2 ± 0.5	1.4 ± 0.6^∗^	1.2 ± 0.4^∗^	1.2 ± 0.3^∗^

ScvO_2T_ [%]	84.6 ± 6.5	85.7 ± 5.6	86.8 ± 4.8	76.9 ± 4.9^∗^	72.7 ± 8.3^∗^	72.6 ± 9.0^∗^
ScvO_2L_ [%]	87.0 ± 5.4	87.3 ± 4.9	87.5 ± 4.6	76.8 ± 4.9^∗^	74.8 ± 2.9^∗^	74.0 ± 6.7^∗^
ScvO_2E_ [%]	82.9 ± 6.7	84.6 ± 6.0	86.3 ± 5.1	77.0 ± 5.1	71.7 ± 10^∗^	72.0 ± 10.1^∗^

BE_T_ [mmol/L]	−2.5 ± 2.9	−5.1 ± 3.3^∗^	−6.3 ± 3.3^∗^	−5.5 ± 2.6^∗^	−5.8 ± 2.1^∗^	−5.5 ± 1.9^∗^
BE_L_ [mmol/L]	−1.9 ± 2.9	−3.2 ± 2.6^∗^	−4.5 ± 2.0^∗^	−4.4 ± 1.8^∗^	−4.7 ± 2.3^∗^	−4.7 ± 2^∗^
BE_E_ [mmol/L]	−2.9 ± 3.0	−6.3 ± 3.1^∗^	−7.5 ± 3.5^∗^	−6.3 ± 2.8^∗^	−6.4 ± 1.9^∗^	−5.8 ± 1.9

Hb_T_ [mg/dL]	10.5 ± 1.7	9.1 ± 1.9^∗^	8.5 ± 1.5^∗^	9.0 ± 1.5^∗^	8.8 ± 1.4^∗^	8.7 ± 1.4^∗^
Hb_L_ [mg/dL]	11.0 ± 1.8	9.3 ± 2.0^∗^	8.8 ± 1.8^∗^	8.8 ± 1.9^∗^	8.4 ± 2.0^∗^	8.2 ± 1.8
Hb_E_ [mg/dL]	10.2 ± 1.6	9.0 ± 1.8	8.3 ± 1.3^∗^	9.2 ± 1.2	9.0 ± 1.1^∗^	9.0 ± 1.0^∗^

**Table 4:** Hemodynamic parameters and oxygenation. EVLWI: extravascular lung water index; CI: cardiac index; AP mean: mean arterial pressure; CVP: central venous pressure; GEDI: global enddiastolic volume index; SVI: stroke volume index; compliance: pulmonary compliance; NE: norepinephrine administration; ^∗^difference to BL in analysis of variance (ANOVA) (*P* < 0.05). BL: directly after induction of anesthesia; OLVimpl15: 15 minutes after beginning OLV; OLVterm15: 15 minutes after cessation of OLV; 6postop: 6 hours after surgery; 12postop: 12 hours after surgery; 24postop: 24 hours after surgery.

	BL	OLVimpl15	OLVterm15	6postop	12postop	24postop
EVLWI_T_ [mL/kg]	7.8 ± 2.5	8.4 ± 3.9	8.5 ± 2.5	8.2 ± 2.9	8.7 ± 2.6	8.1 ± 2.4
EVLWI_L_ [mL/kg]	7.9 ± 1.7	8.1 ± 3.2	8.5 ± 2.5	7.8 ± 2.4	8 ± 1.8	7.2 ± 1.9
EVLWI_E_ [mL/kg]	7.8 ± 3	8.5 ± 3.4	8.6 ± 2.6	8.5 ± 3.1	8.98 ± 3	9.1 ± 2.5

p_a_O_2_/F_i_O_2_-ratio_T_ [mmHg]	419 ± 122	186 ± 94^∗^	334 ± 92^∗^	n.d.	n.d.	n.d.
p_a_O_2_/F_i_O_2_-ratio_L_ [mmHg]	462 ± 140	202 ± 105^∗^	338 ± 112^∗^	n.d.	n.d.	n.d.
p_a_O_2_/F_i_O_2_-ratio_E_ [mmHg]	389 ± 101	174 ± 87^∗^	332 ± 80	330 ± 111	329 ± 105	303 ± 74^∗^

CI_T_ [L/min/m^2^]	2.8 ± 0.9	3.6 ± 0.9^∗^	3.5 ± 0.8	3.5 ± 0.9^∗^	3.6 ± 0.9	3.5 ± 0.9
CI_L_ [L/min/m^2^]	3 ± 0.9	3.4 ± 0.66	3.5 ± 0.8	3.6 ± 1.1	3.9 ± 1.2	3.6 ± 1.2
CI_E_ [L/min/m^2^]	2.7 ± 0.9	3.7 ± 1^∗^	3.5 ± 0.7	3.5 ± 0.6	3.5 ± 0.7	3.4 ± 0.6

AP mean_T_ [mmHg]	78.7 ± 15	74.9 ± 14.1	73 ± 12.1	78.9 ± 14.1	73.9 ± 16.1	76.6 ± 8.2
AP mean_L_ [mmHg]	84.8 ± 13.3	84.7 ± 12.6	80.1 ± 14.1	85.9 ± 11.7	81.3 ± 14.2	81.4 ± 6.9
AP mean_E_ [mmHg]	74.5 ± 15	68.2 ± 11	68.1 ± 7.8	74 ± 13.7	69.5 ± 15.9	73.8 ± 7.7

CVP_T_ [mmHg]	7.4 ± 2	7.3 ± 2.9	6.6 ± 2.7	6.1 ± 2	5.8 ± 2.4	6 ± 2.2
CVP_L_ [mmHg]	7.9 ± 0.3	7.2 ± 2.5	6.4 ± 2.3	5.9 ± 2.2	5.4 ± 1.2	5.5 ± 1.5
CVP_E _ [mmHg]	7 ± 2.6	7.4 ± 3.3	6.8 ± 3	6.3 ± 1.9	6 ± 2.9	6.2 ± 2.6

GEDI_T _ [mL/m^2^]	673 ± 169	642 ± 152	633 ± 144	649 ± 114	665 ± 124	658 ± 123
GEDI_L_ [mL/m^2^]	702 ± 167	694 ± 121	689 ± 170	670 ± 93	657 ± 138	640 ± 101
GEDI_E _ [mL/m^2^]	653 ± 173	607 ± 165	594 ± 112	635 ± 128	670 ± 119	669 ± 137

SVI_T_ [mL/m^2^]	40.2 ± 11.1	48.4 ± 12.4	46.9 ± 15.3	49.6 ± 13.6	49.7 ± 13.8	47.3 ± 13.9
SVI_E_ [mL/m^2^]	38.9 ± 10.0	50.2 ± 13.7	46.7 ± 16.1	47.8 ± 12.3	47.1 ± 12.5	46.5 ± 12.8
SVI_L_ [mL/m^2^]	42.0 ± 12.7	46.1 ± 10.4	47.1 ± 14.9	52.0 ± 15.5	53.1 ± 15.3	48.5 ± 15.8

Compliance_T_ [L/cmH_2_O]	0.049 ± 0.013	0.023 ± 0.006^∗^	0.046 ± 0.012	n.d.	n.d.	n.d.
Compliance_L_ [L/cmH_2_O]	0.055 ± 0.014	0.024 ± 0.006^∗^	0.045 ± 0.013	n.d.	n.d.	n.d.
Compliance_E_ [L/cmH_2_O]	0.046 ± 0.01	0.022 ± 0.005^∗^	0.046 ± 0.012	0.039 ± 0.013	0.052 ± 0.023	0.062 ± 0.024

NE_T _ [*μ*g/kg/min]	0.07 ± 0.02	0.12 ± 0.02	0.08 ± 0.01	0.04 ± 0.02	0.03 ± 0.01	0.02^∗^ ± 0.01
NE_L _ [*μ*g/kg/min]	0.05 ± 0.01	0.09 ± 0.02	0.06 ± 0.02	0.01 ± 0.01	0.01 ± 0.01	0.008^∗^ ± 0.01
NE_E _ [*μ*g/kg/min]	0.08 ± 0.01	0.14 ± 0.01	0.09 ± 0.01	0.06 ± 0.02	0.04 ± 0.01	0.03^∗^ ± 0.01
